# An unusually large osteochondroma of the mandibular angle: a case report

**DOI:** 10.1186/s12957-017-1270-9

**Published:** 2017-11-13

**Authors:** Ryosuke Abe, Ikuya Miyamoto, Hirotaka Sato, Daishi Saitou, Genki Yamaya, Hiroyuki Yamada

**Affiliations:** 1 Division of Oral and Maxillofacial Surgery, Department of Oral and Maxillofacial Reconstructive Surgery, School of Dentistry, Iwate Medical University, 19-1 Uchimaru, Morioka, Iwate 020-8505 Japan; 2 Division of Anatomical and Cellular Pathology, Department of Pathology, School of Dentistry, Iwate Medical University, 2-1-1 Nishitokuta, Yahaba-cho, Shiwa-gun, Iwate 028-3694 Japan

**Keywords:** Benign bone tumor-osteochondroma-mandibular angle-cone beam CT

## Abstract

**Background:**

Osteochondroma is a benign bone tumor that can occur in both the mesenchymal and craniofacial bones. However, craniofacial osteochondromas are extremely rare, because the mandible develops by intramembranous ossification rather than by endochondral ossification.

**Case presentation:**

The most common site of craniofacial osteochondroma is the mandibular condyle, followed by the coronoid process. In the present study, we have described the case of a 64-year-old Japanese man with an unusually large osteochondroma located on the internal angle of the mandibular body. Clinical, radiological, pathological, and treatment-related aspects are discussed with respect to the tumor origins.

**Conclusions:**

In the medical literature, there have been few reports of large osteochondromas of the mandibular angle with no clinical symptoms.

## Background

Osteochondroma (OC) is the most common benign neoplasms of the skeleton, comprising 35.8% of all benign bone tumors, according to a study by Lim et al. [1]. However, OC of the craniofacial bones is extremely rare. In 1899, Jacob was the first to describe an OC of the coronoid process; for this reason, this particular condition has been named “Jacob’s disease.” In this seminal study, the tumor formed a pseudoarthrotic joint between the coronoid process and the zygomatic arch [2]. In the craniofacial region, the most common site of OC is the mandibular condyle, followed by coronoid process. In both cases, the tumor can result in morphological and functional disturbances.

Herein, we report an extremely rare, voluminous OC of the mandibular angle that showed no clinical symptoms. To the best of our knowledge, only four cases have been reported involving OC of the mandibular angle; moreover, the present case was unique, because never before has such a large OC been reported at the mandibular angle that was continuous with the mandibular inferior body and that presented no clinical symptoms.

## Case presentation

**Fig. 1:**
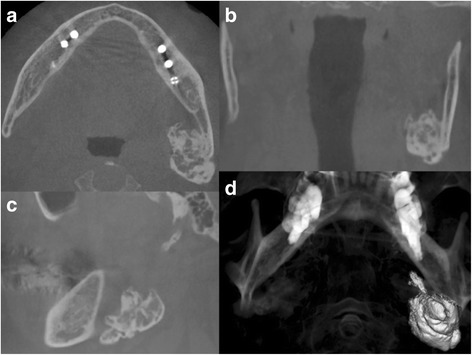
**a**–**c** Axial, coronal, and sagittal CBCT scan showing the lesion attached to the inner surface of the mandible. **d** Three-dimensional cone beam CT image showing an irregular bony mass with an outgrowth at the inferior border of the mandibular angle and extending to the submandibular area. The mass measures 33.3 × 41.0 × 20.2 mm

A 64-year-old Japanese man presented to a private dental clinic for dental caries treatment; a routine, panoramic radiograph showed a mixed radiolucent and radiopaque mass on the left side of the mandibular angle. The dentist suggested that the patient seek further examination and referred him to our university hospital. The patient had no history of trauma or surgery in the left mandibular area, and he stated that none of his family members had any similar bony outgrowths. He had a medical history of controlled prostate cancer and atrial fibrillation, but hematological and biochemical examinations did not reveal any pathological findings. His facial appearance was symmetrical, with mild swelling at the left inferior border of the mandibular angle. There was no swelling or tenderness in the submandibular lymph nodes, and the patient’s intraoral condition showed no remarkable findings. Nonetheless, three-dimensional cone beam computed tomography revealed a mixed radiolucent and radiopaque mass at the left-side inferior mandibular angle and submandibular region (Fig. 1). The mass measured 33.3 × 41.0 × 20.2 mm. On the basis of these clinical signs, the patient was diagnosed as having a peripheral osteoma of the mandible, and a surgical resection of bony mass was performed under general anesthesia.

**Fig. 2:**
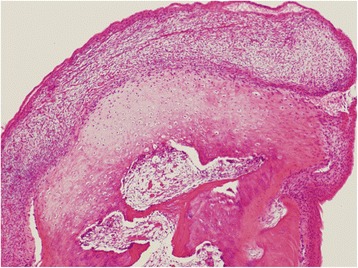
Histopathological section showing layers of thick perichondrium, cartilage, and bone with bone marrow tissue (hematoxylin and eosin; original magnification × 40)

Specifically, a submandibular incision was made and the periosteum was elevated, exposing the mass, which was then lobulated without any adhesive periosteum. The mass was attached to the inferior border of the mandibular angle, and it extended from the internal mandibular angle to the front of the anterior mandibular ramus. The tumor was cut from the mandibular inferior body using a fissure bur; the excised specimen was a hard, ovular mass with a smooth, opalescent surface and without capsules. Histopathologically, the tumor showed a cartilage-capped bony projection with bone marrow that was covered by a thick perichondrium (Fig. 2). The post-operative course was uneventful, and the patient showed no signs of recurrence during a 1-year follow-up period.

## Discussion

Currently, the World Health Organization defines OC as “a cartilage-capped bony projection arising on the external surface of bone containing a marrow cavity that is continuous with that of the underlying bone” [3]. OCs of the mandibular region are rare, with 38 cases of OC involving the mandibular condyle, 49 involving the coronoid process, and a handful involving the maxilla reported [4–6]. Moreover, OCs of the mandibular body are extremely uncommon; indeed, to the best of our knowledge, only four cases of OC of the mandibular angle, other than our own, have been reported, and the present case represents the largest such tumor ever reported [7].

The main reason OCs of the mandibular angle are so rare is because the mandibles develop by intramembranous ossification rather than by endochondral ossification [8]. In this regard, the etiology of OCs—and even their precise disease classification—is controversial; that is, they may be true tumors, developmental abnormalities, or manifestations of a response to trauma or infection. In this regard, to reflect the diverse activities of OCs, a multitude of descriptive terms have been applied. For instance, some authors have termed these lesions “osteocartilaginous exostoses,” which implies that some kind of reactive exostosis has occurred. Conversely, others have dubbed them “osteochondromas,” considering them a true bone tumor [9].

With regard to the cause of OCs, two possible etiologies have been proposed. The first, advanced by Lichtenstein, is that OCs result from induced or spontaneous metaplasia of the periosteum, which has the pluripotentiality to yield cartilage cells as precursors. These cells then form an OC through endochondral ossification [10]. According to the second explanation, a low-grade, chronic inflammatory reaction caused by an odontogenic infection leads to OC. More specifically, infection and inflammation may trigger remnants of the embryological Meckel cartilage and accessory cartilaginous nodules, resulting in excessive growth and, ultimately, OC development.

In the present case, the mass was located a considerable distance from the patient’s teeth or implants; for this reason, it seems unlikely that the tumor resulted from chronic inflammation.

OC needs to be distinguished histologically from osteoma, benign osteoblastoma, chondroma, giant cell tumor, and chondroblastoma [6, 11, 12]. Additionally, unilateral submandibular masses may be attributed to a number of pathological conditions of various origins [13]. In the differential diagnosis of the present case, lymph node calcification and sialolithiasis of the submandibular salivary gland were also considered. Lymph node tuberculosis is most commonly encountered in cervical lymph nodes [14].

The accepted modality of treatment for OC is local excision of the tumor and surrounding bone, and recurrence is rare. Insufficient excision causes tumor recurrence [15]. Moreover, a small proportion of cases (less than 2%) do lead to recurrence or malignant transformation [16]. The most common malignant tumors in such cases are low-grade chondrosarcomas, which tend to arise in the cartilage cap, as well as osteosarcomas, which grow at the base of the OC.

Although our patient experienced no recurrence, the follow-up time in the present study was insufficient to exclude the possibility of a malignant transformation; therefore, further follow-ups are mandatory.

## Conclusions

In the medical literature, there have been few reports of large osteochondromas of the mandibular angle with no clinical symptoms and the present case represents the largest such tumor ever reported.

## Abbreviations

OC: Osteochondroma
